# Global research trends in therapeutic drug monitoring of antimicrobials from 2000 to 2023: a bibliometric analysis

**DOI:** 10.3389/fphar.2024.1474878

**Published:** 2024-12-03

**Authors:** Hao Li, Manxue Jiang, Lingti Kong

**Affiliations:** ^1^ Department of Pharmacy, The First Affiliated Hospital of Bengbu Medical University, Bengbu, China; ^2^ School of Pharmacy, Bengbu Medical University, Bengbu, China; ^3^ Institute of Emergency and Critical Care Medicine, The First Affifiliated Hospital of Bengbu Medical University, Bengbu, China

**Keywords:** bibliometrics, citespace, VOSviewer, antimicrobial, therapeutic drug monitoring

## Abstract

**Objective:**

The practice of therapeutic drug monitoring (TDM) is widely used for maximizing the clinical efficacy of antimicrobials. However, a systematic bibliometric analysis providing an overview of this field is lacking at present. The aim of the current study was to identify hotspots and trends in antimicrobial TDM, highlight collaborations and influences among countries, institutions, and journals, and assess the knowledge base for further development of clinical research.

**Research Design and Methods:**

Articles and reviews related to TDM of antimicrobials from the Web of Science Core Collection were collected. CiteSpace and VOSviewer, two visualization tools, were utilized to graphically assess the key elements within this domain, including mapping of countries and regions, institutions, keywords, and references associated with the field of antimicrobial TDM. Through this approach, we were able to successfully provide a comprehensive visual overview of the research landscape, highlighting the significant players and thematic trends in the literature.

**Results:**

From 2000 to 2023, a total of 17,236 authors from 4,112 institutions in 112 countries/regions published 3,710 papers in 819 academic journals. The United States had the highest number of publications, with University of Queensland identified as the most active institution. The journal with the greatest number of publications was Therapeutic Drug Monitoring, whereas Antimicrobial Agents and Chemotherapy was the most co-cited journal. Current research focuses on pharmacokinetics, pharmacodynamics, vancomycin, posaconazole, invasive fungal infection and critically ill patients. Promising hotspots for future research include vancomycin, voriconazole, meropenem, isavuconazole, posaconazole, and teicoplanin. Moreover, vancomycin and critically ill patients remain a hot topic of future research.

**Conclusion:**

Using bibliometric and visualization methods, the research hotspots of antimicrobial drugs in TDM were analyzed. The continued increase in the number of annual publications demonstrates the vital significance of TDM for antimicrobials. Data from this study provide a valuable reference for future research trends in TDM of antimicrobial agents.

## 1 Introduction

Antibacterial drugs are generally defined as agents that exert bactericidal or bacteriostatic effects by inhibiting bacterial growth or vital cellular functions (Doyle and Stephens, 2019). While the importance of antimicrobials in modern medicine cannot be overemphasized (Devasahayam et al., 2010; Strzelecka and Świątek, 2021), antibiotic resistance has emerged as one of the most serious global public health threats of this century (Kalın et al., 2023; Prestinaci et al., 2015). The golden age of antibiotic discovery from the 1940s through the 1960s has been succeeded by a dramatic increase in antibiotic resistance (Abdul-Aziz et al., 2020; Devasahayam et al., 2010; Repac Antić et al., 2022). Thus, the process of therapeutic drug monitoring (TDM) is extremely beneficial for determining the most effective dose of antibiotics to ensure the appropriate delivery of antimicrobial agents for treatment purposes (Kalın et al., 2023; Lesko and Schmidt, 2012; Pistolesi et al., 2019; Veiga and Paiva, 2018).

The core component of TDM involves monitoring the drug concentration in a patient’s body to ensure that a safe and effective range is maintained throughout the course of therapy (Gallerani et al., 2023; Hussain et al., 2021; Jang et al., 2016). This helps to ensure that the optimal therapeutic window of the drug is achieved during treatment, which improves treatment efficacy (Roberts et al., 2014) and reduces the risk of drug-induced toxicity in patients. The implementation of TDM is more necessary in complex cases of drug-drug interactions or patient comorbidities (Jian et al., 2023; Micaglio et al., 2021; Matsumoto et al., 2022; Kang and Lee, 2009). Some antimicrobials have a narrow therapeutic window and significant individual variations in subtherapeutic doses of antibiotics can lead to poor patient prognosis and increased likelihood of the emergence of drug-resistant strains of bacteria (Xavier et al., 2023; Penson and McCloskey, 2023). TDM is therefore particularly critical to ensure the optimal utilization of antimicrobial agents.

Bibliometrics is a contemporary approach for systematic evaluation of a specific area that employs Mathematics and Statistics as tools for assessing earlier research (Blakeman, 2018). The advantage of bibliometrics over other methods, such as traditional reviews, meta-analyses, or empirical studies, is that it enables researchers to rapidly identify the hotspots and trends in a particular field of study (Hou et al., 2022). The present study explores the advances in TDM of antimicrobial drugs from a bibliometric perspective, with the aim of understanding the dynamics and emerging trends of antimicrobial drug research and providing a comprehensive perspective for clinicians and pharmacy researchers in this sector.

## 2 Materials and methods

### 2.1 Data collection

**FIGURE 1:**
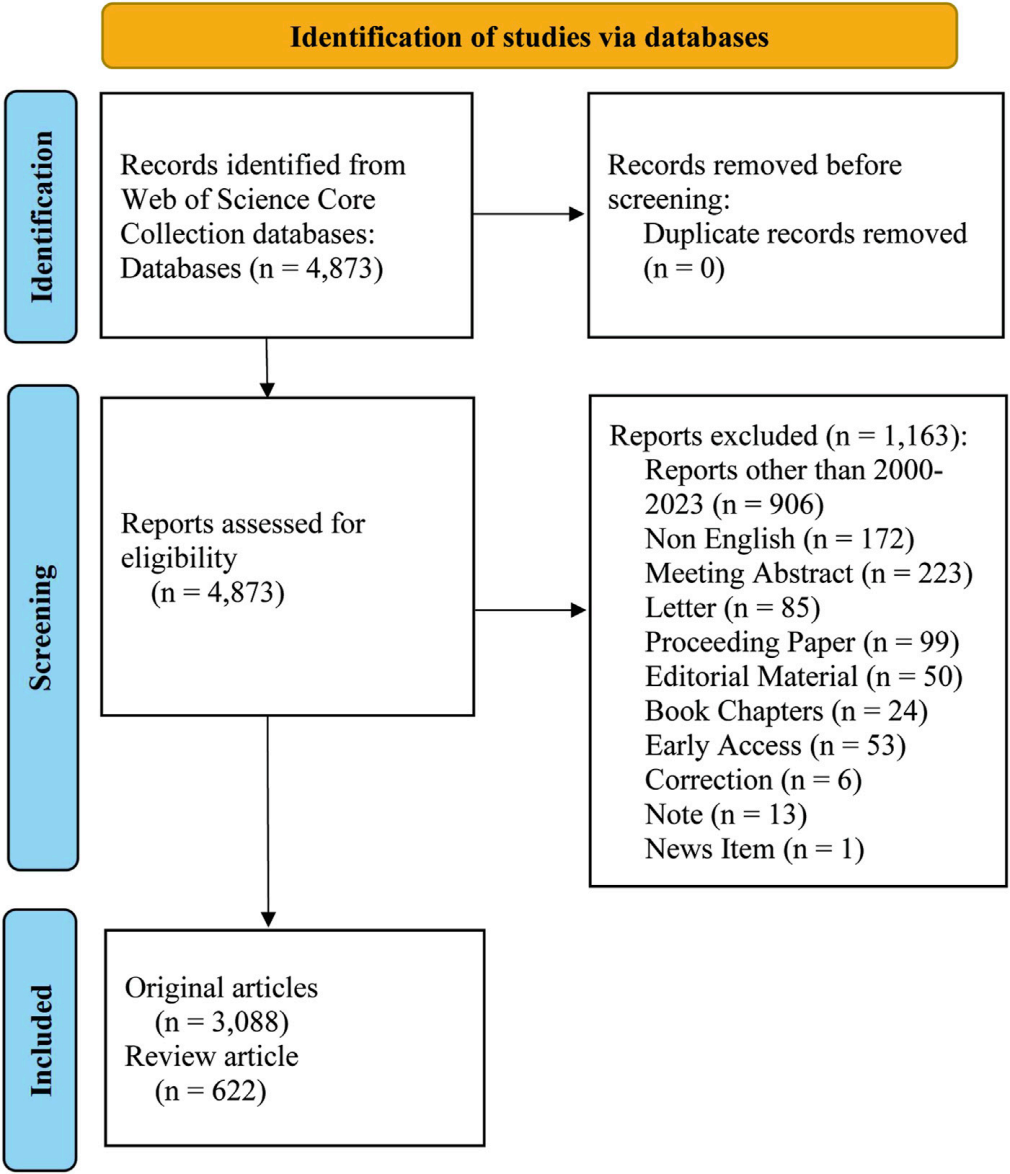
Flow chart of literature screening.

Web of Science (WOS) is a highly utilized scholarly database containing over 12,000 influential journals (Wu et al., 2021b) and widely acknowledged as the most comprehensive and reliable database for bibliometric analysis compared to other resources, such as Scopus and PubMed (Sun et al., 2022). Previous studies have shown that Web of Science Core Collection database (WOSCC) is the most suitable repository for bibliometric studies among those that fulfill the requirements for global-level analysis (Deng et al., 2020; Yeung, 2019). In the current study, the relevant literature was searched and exported on 17 January 2024. The time period considered was 1 January 2000 to 31 December 2023, when selected papers and review papers were downloaded. Meeting Abstract, Early Access, Book Chapters, Editorial Material, Proceeding Paper, Letter, Correction, Note and News Item were excluded. The language restriction was English. A total of 4,873 articles were retrieved, among which 1,163 were excluded. The remaining 3,710 articles were exported in plain text with the file name “download_txt” (Figure 1).

The database advanced search entry is set as the following: TS = (“TDM” OR “Drug Monitoring” OR “Monitoring Drug” OR “Therapeutic Drug Monitoring” OR “Drug Monitoring Therapeutic” OR “Monitoring Therapeutic Drug” OR “target concentration intervention” OR “target concentration strategy” OR “model-based precision dosing”) AND TS = (“Anti-Bacterial Agents” OR “Agents Anti-Bacterial” OR “Anti Bacterial Agents” OR “Antibacterial Agents” OR “Agents Antibacterial” OR “Antibacterial Agent” OR “Agent Antibacterial” OR “Anti-Bacterial Compounds” OR “Anti Bacterial Compounds” OR “Compounds Anti-Bacterial” OR “Anti-Bacterial Agent” OR “Agent Anti-Bacterial” OR “Anti Bacterial Agent” OR “Anti-Bacterial Compound” OR “Anti Bacterial Compound” OR “Compound Anti-Bacterial” OR “Bacteriocidal Agents” OR “Agents Bacteriocidal” OR “Bacteriocidal Agent” OR “Agent Bacteriocidal” OR “bactericide” OR “Anti-Mycobacterial Agents” OR “bacteriocines” OR “Agents Anti-Mycobacterial” OR “Anti Mycobacterial Agents” OR “Anti-Mycobacterial Agent” OR “Agent Anti-Mycobacterial” OR “Anti Mycobacterial Agent” OR “Antimycobacterial Agent” OR “Agent Antimycobacterial” OR “Antimycobacterial Agents” OR “Agents Antimycobacterial” OR “Antibiotic” OR “Antibiotics” OR “Infections” OR “Infection” OR “meropenem” OR “vancomycin” OR “piperacillin” OR “ceftazidime” OR “cefepime” OR “gentamicin” OR “tazobactam” OR “piperacillin-tazobactam” OR “ciprofloxacin” OR “amikacin” OR “ceftriaxone” OR “daptomycin” OR “ertapenem” OR “imipenem” OR “moxifloxacin” OR “voriconazole” OR “colistin” OR “flucloxacillin” OR “tobramycin” OR “clarithromycin” OR “amoxicillin” OR “levofloxacin” OR “cilastatin” OR “polymyxin-b” OR “teicoplanin” OR “trimethoprim-sulfamethoxazole” OR “adriamycin” OR “amphotericin-b” OR “ampicillin” OR “cefotaxime” OR “carbapenems” OR “caspofungin” OR “ceftaroline fosamil” OR “cefazolin”).

### 2.2 Data analysis and visualization

CiteSpace is a web-based Java application for analysis and visualization of co-citation networks (Chen, 2004). CiteSpace research uses information contained in the articles to evaluate and predict future developments in the field (Liu et al., 2019). The main distinguishing feature of CiteSpace is the use of a diverse, time-phased and dynamic visualization language for citation analysis, showing evolution of the field on a knowledge map of the citation network through a clever spatial layout (Chen et al., 2010). The automatic identification of research frontiers with citation node literature and co-citation clustering as the knowledge base reflects the interpretability of the graph. In this study, CiteSpace 6.3.R1 (Advanced) was used to analyze and visualize the research hotspots and evolution of antimicrobial drugs in TDM since the twenty-first century and predict future trends in the field.

VOSviewer presents a means to construct author or journal maps based on citation data or keyword maps based on co-occurrence data (Wu et al., 2022). The program provides a viewer that enables detailed examination of bibliometric maps. VOSviewer can display maps in numerous ways that highlight different aspects (van Eck and Waltman, 2010), including network, coverage, and density maps, with each focusing on a distinct dimension. The features of VOSviewer are particularly well suited for displaying large bibliometric charts in an understandable format (van Eck and Waltman, 2010). We initially analyzed the major journals, co-cited journals, and co-occurring keywords according to the WOS data using VOSviewer 1.6.20 and subsequently created related network and density maps. The structure of scientific research, research hotspots, and development trends in this field were identified through relevant analyses.

## 3 Results

### 3.1 Trends in publications from 2000 to 2023

**FIGURE 2:**
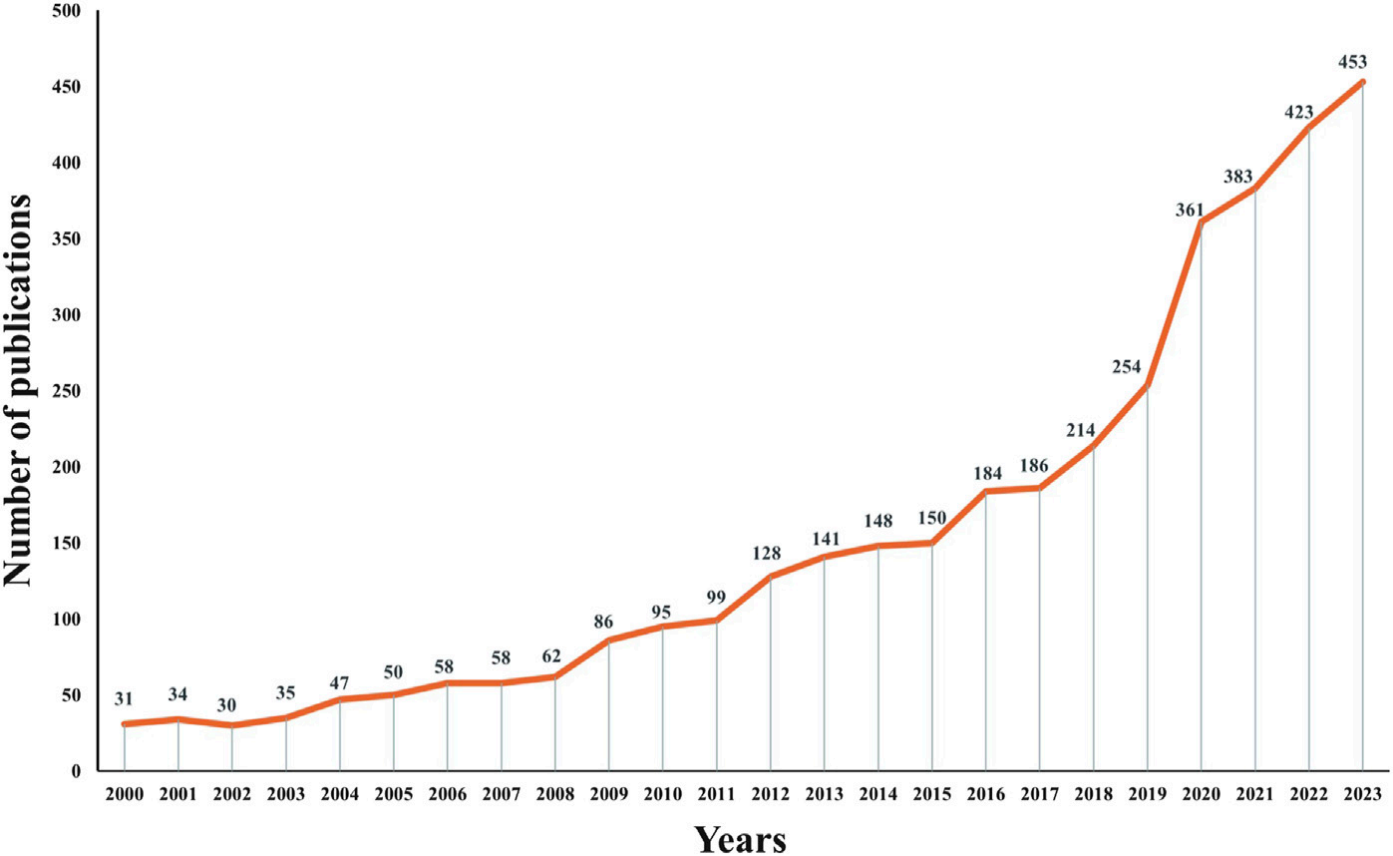
Trends in the number of publications from the twenty-first century in studies of therapeutic drug monitoring for antimicrobial drug applications.

A total of 3,710 relevant papers published between 2000 and 2023 were collected, the number of articles related to antimicrobial drugs and TDM showed a clear upward trend, indicating that this topic continues to attract considerable research attention (Figure 2). Among these, the maximum number of 453 articles were published in 2023.

### 3.2 Article type and specific drug type

**FIGURE 3:**
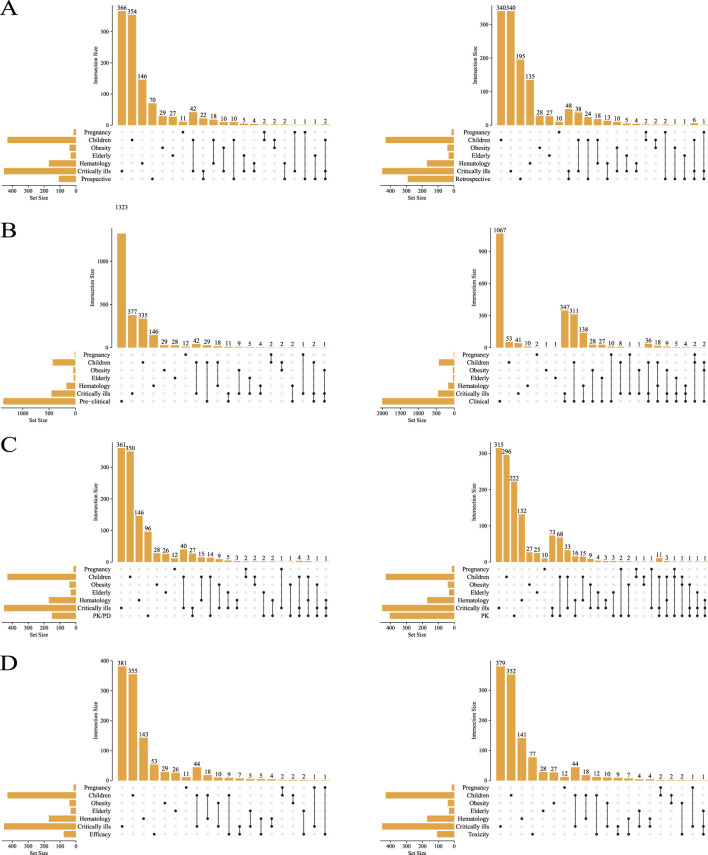
The types of studies included were compared with the types of people involved. Prospective vs. retrospective **(A)**, preclinical vs. clinical **(B)**, PK/PD vs. PK **(C)**, toxicity vs. efficacy **(D)**.

All the included studies were summarized according to study type and population type, as shown in Figure 3. The number of prospective articles (n = 108) was less than that of retrospective articles (n = 292), both of which focus more on critically ill patients and children (Figure 3A). There are more PK articles (n = 406) than PK/PD articles (n = 151) (Figure 3C), and more articles on safety (n = 109) than efficacy (n = 77) (Figure 3D).

**FIGURE 4:**
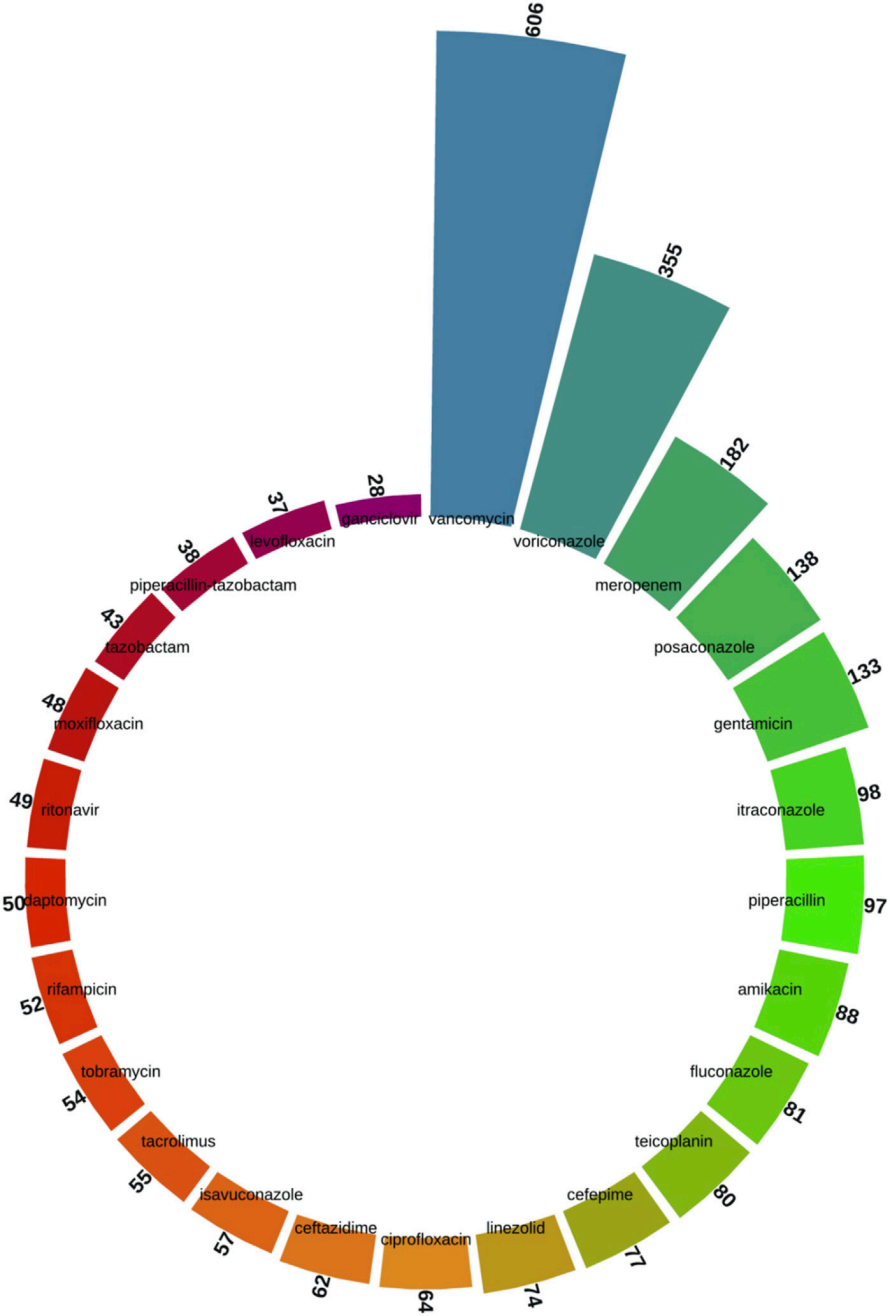
Top 25 drug-specific keywords screened from the keyword co-occurrence network of antimicrobial drugs in therapeutic drug monitoring studies.

As shown in Figure 4, we screened the top 25 specific drug species from all the keywords. Vancomycin (n = 606), voriconazole (n = 355), meropenem (n = 182), posaconazole (n = 138), gentamicin (n = 133), itraconazole (n = 98), piperacillin (n = 97), amikacin (n = 88), fluconazole (n = 81), and teicoplanin (n = 80) were among the top drug-related keywords.

### 3.3 Countries/regions and institutions

**FIGURE 5:**
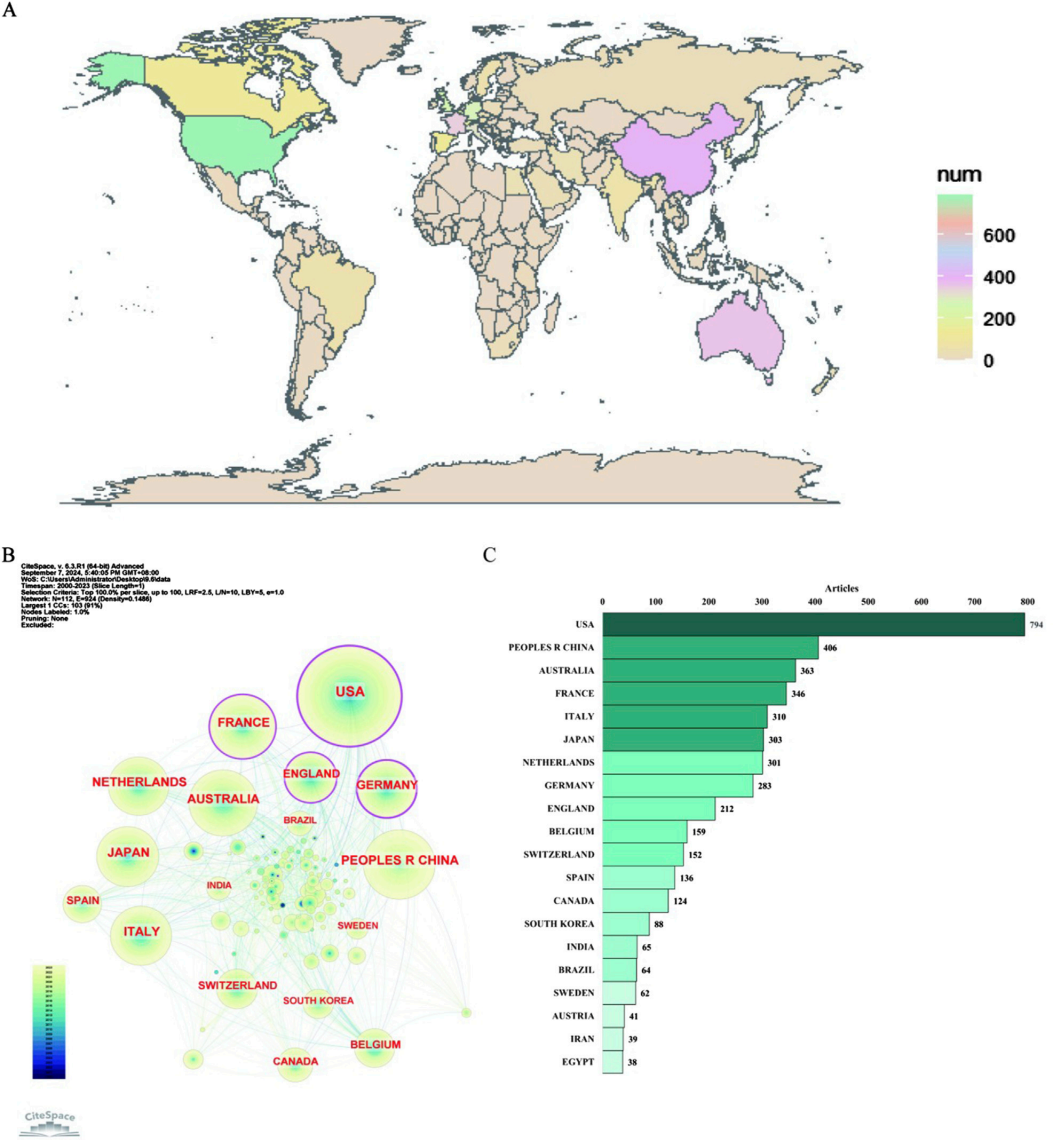
Distribution of publications on antimicrobials in therapeutic drug monitoring research: national geographic distribution map **(A)**, country collaborative network map **(B)** and top 20 countries **(C)**.

The countries studied in this field geographically span six continents, notably: North America (USA, Canada), Asia (China, Japan, Korea, India, Iran), Oceania (Australia), Europe (France, Italy, Netherlands, Germany, England, Belgium, Switzerland, Spain, Sweden, Austria), South America (Brazil), and Africa (Egypt), as shown in Figure 5A.

As shown in Figure 5B, the tree wheel history represents the record of published articles from a given country. Different colors of the tree wheel represent the corresponding time and the overall size reflects the number of publications originating from the country. The United States, France, England, and Germany are displayed as purple outer rings, which are characterized by a high degree of centrality (≥0.1) and often considered to be important turning points leading to revolutionary discoveries. Figure 5C displays the number of articles per country/region for the top twenty countries. The United States had the highest number of publications (n = 794), followed by China (n = 406), Australia (n = 363), France (n = 346), Italy (n = 310), Japan (n = 303), the Netherlands (n = 301), Germany (n = 283), and England (n = 212), among others.

**FIGURE 6:**
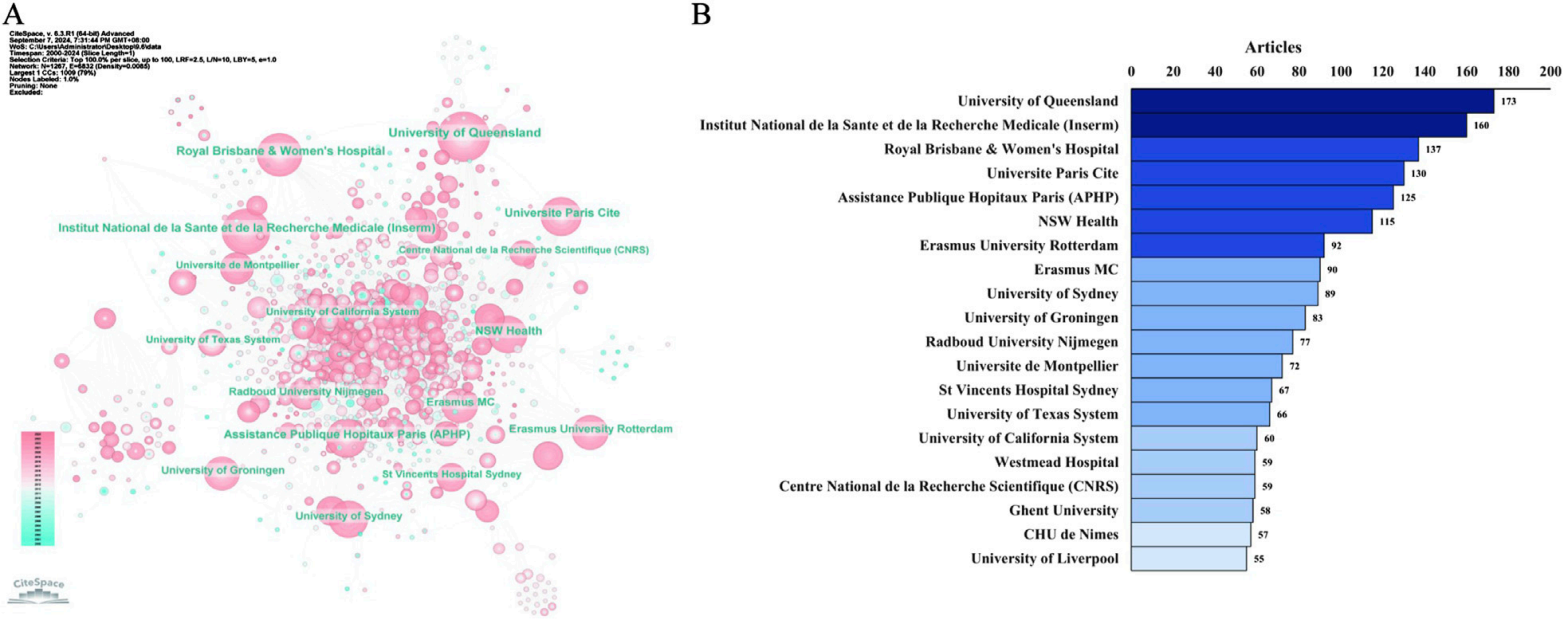
Distribution of publications on antimicrobials in therapeutic drug monitoring research: a network map of institutional collaborations **(A)** and the top 20 institutions **(B)**.

We further observed the network of institutional collaborations on the topic of TDM of antimicrobial drugs (Figure 6A). Among the top 20 institutions (Figure 6B), the highest number of publications was produced by the University of Queensland (n = 173), followed by Institut National de la Sante et de la Recherche Medicale (Inserm) (n = 160), Royal Brisbane & Women’s Hospital, the Paris City University (n = 137), Universite Paris Cite (n = 130), Assistance Publique Hopitaux Paris (APHP) (n = 125), NSW Health (n = 115), etc.

**FIGURE 7:**
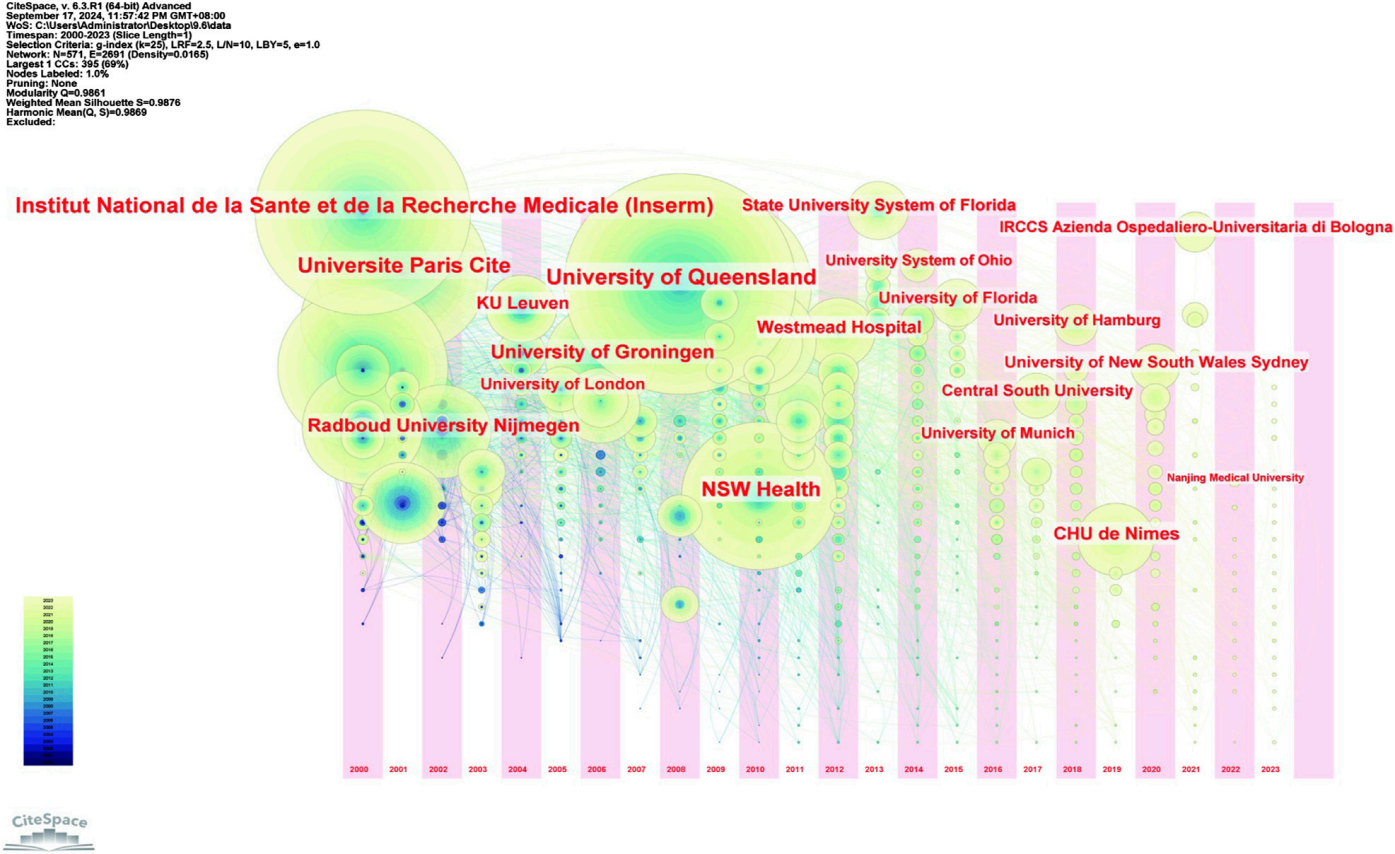
The difference of the number of institutional publications over time. Notes: The horizontal axis of the institution represents the year in which it first published research in this field, different colours in the circle represent different years, and the size of the circle represents the cumulative frequency of publications.

In Figure 7, a time series graph was created based on the number of agency publications. Institut National de la Sante et de la Recherche Medicale (France), University of Queensland (Australia), and CHU de Nimes (France), the number of publications from these three institutions is constantly increasing, and there will still be sustained output in this field in the future.

### 3.4 Major and Co-cited journals

A total of 3,710 articles published in 819 journals were identified. The names of the top 10 journals in terms of number of publications are presented in Table 1. *Therapeutic Drug Monitoring* had the highest publication rate, followed by *Antimicrobial Agents and Chemotherapy*, *Journal of Antimicrobial Chemotherapy*, *International Journal of Antimicrobial Agents*, and *Antibiotics-Basel*. Among the top 10 journals, six belonged to JCR Q1.

**TABLE 1:** Top 10 journals on antimicrobials in therapeutic drug monitoring research.

Rank	Journal	Count (%)	IF (2023)	JCR
1	Therapeutic Drug Monitoring	236 (6.36%)	2.8	Q2
2	Antimicrobial Agents and Chemotherapy	169 (4.56%)	4.1	Q1
3	Journal of Antimicrobial Chemotherapy	145 (3.91%)	3.9	Q1
4	International Journal of Antimicrobial Agents	103 (2.78%)	4.9	Q1
5	Antibiotics-Basel	99 (2.67%)	4.3	Q1
6	Journal of Pharmaceutical and Biomedical Analysis	70 (1.89%)	3.1	Q2
7	Journal of Chromatography B-Analytical Technologies in The Biomedical and Life Sciences	61 (1.64%)	2.8	Q2
8	British Journal of Clinical Pharmacology	59 (1.59%)	3.1	Q2
9	Frontiers in Pharmacology	55 (1.48%)	4.4	Q1
10	Clinical Pharmacokinetics	54 (1.46%)	4.6	Q1

In the analysis of co-cited journals, 21 had more than 1,000 co-citations out of 12,733 journals. Table 2 displays the top 10 journal names in terms of number of cited papers. *Antimicrobial Agents and Chemotherapy* had the highest number of co-citations, two of which had an IF ≥ 5, with six journals belonging to JCR Q1.

**TABLE 2:** Top 10 co-cited journals for antimicrobials in therapeutic drug monitoring research.

Rank	Co-cited journal	Co-citation	IF (2023)	JCR
1	Antimicrobial Agents and Chemotherapy	14,220	4.1	Q1
2	Clinical Infectious Diseases	7,666	8.2	Q1
3	Journal of Antimicrobial Chemotherapy	7,173	3.9	Q1
4	International Journal of Antimicrobial Agents	3,589	4.9	Q1
5	Clinical Pharmacokinetics	3,434	4.6	Q1
6	Therapeutic Drug Monitoring	3,393	2.8	Q2
7	British Journal of Clinical Pharmacology	2,252	3.1	Q2
8	Journal of Chromatography B-Analytical Technologies in The Biomedical and Life Sciences	2,149	2.8	Q2
9	Pharmacotherapy	1,894	2.9	Q2
10	Clinical Pharmacology & Therapeutics	1,874	6.3	Q1

**FIGURE 8:**
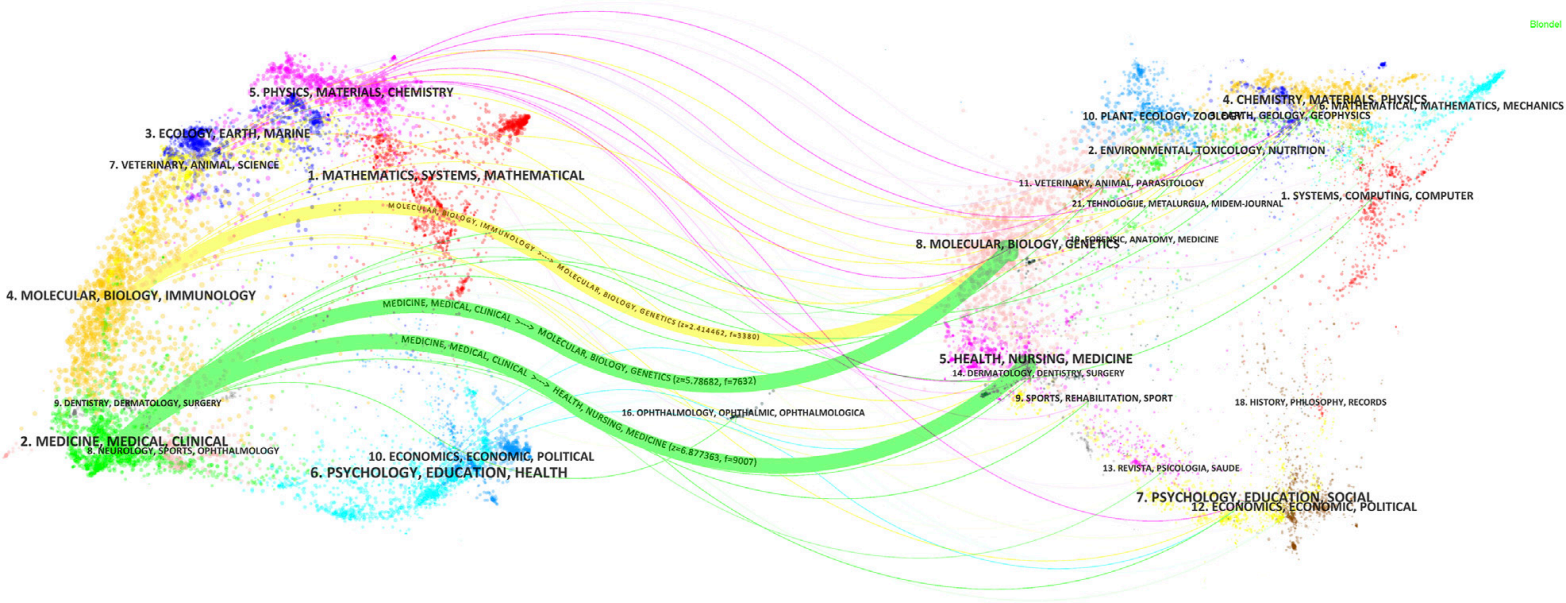
Double-map coverage of journals related to antimicrobials in therapeutic drug monitoring research. Note: Citing journals are on the left, cited journals are on the right, and colored paths indicate citation relationships.

The Journal biplot overlay depicts the distribution of topics in academic journals, with citing journals displayed on the left and cited journals on the right (Figure 8). The different colored paths indicate the citation relationship. Two green and one yellow citation paths were mainly identified. Citing journals are known as ‘research frontiers’ and cited journals as ‘knowledge bases’. As shown in Figure 8, articles published in Molecular/Biology/Immunology journals primarily cited articles from Molecular/Biology/Genetics journals while articles published in Medicine/Medical/Clinical journals mainly cited articles from Molecular/Biology/Genetics and Health/Nursing/Medicine journals.

### 3.5 Keyword Co-occurrence and clusters

**FIGURE 9:**
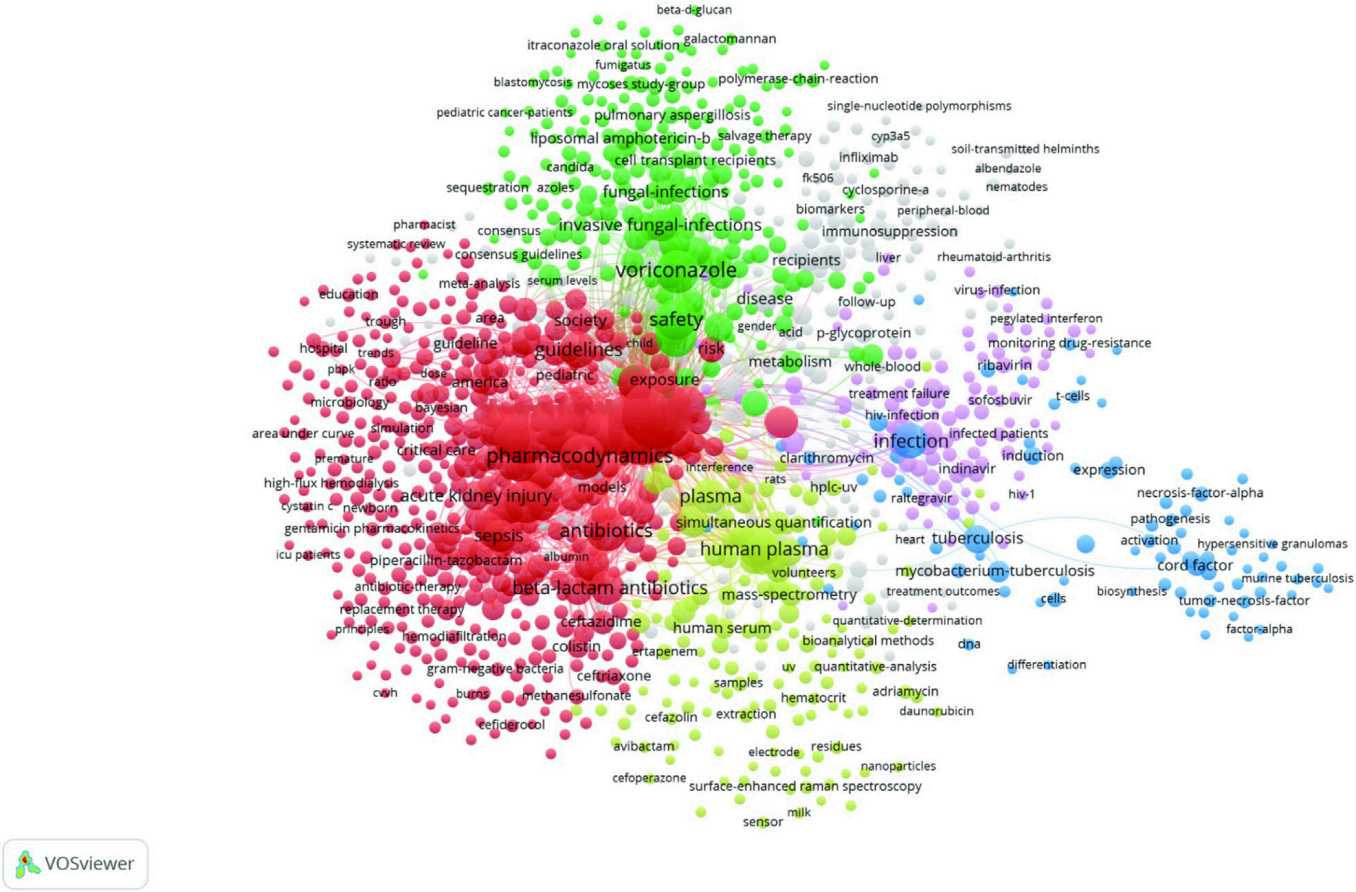
Keyword co-occurrence network of antimicrobial drugs in therapeutic drug monitoring studies. Notes: Node and word sizes reflect co-occurrence frequency, links indicate co-occurrence relationships, and the same node color indicates the same cluster.

Keyword co-occurrence analysis revealed a total of 9,180 keywords, with 1,232 keywords co-occurring ≥5 times that were utilized to generate a keyword co-occurrence map (Figure 9).

In the network analysis diagram, keywords were divided into five clusters, with high correlations within each cluster (Figure 9). The largest clusters, shown in red, comprised 456 terms with a co-occurrence of ≥300 and were related to therapeutic drug monitoring, pharmacokinetics, vancomycin, pharmacodynamics, antibiotics and critically-ill patients. The second cluster is green and contains 220 terms with 7 co-occurrences ≥100, from highest to lowest being voriconazole, safety, management, invasive fungal-infections, posaconazole and aspergillosis. The third cluster is yellow, which contains 155 terms, with 6 co-occurrences ≥100, from highest to lowest being plasma, human plasma, performance liquid-chromatography, serum, quantification, validation. The fourth cluster is pink and contains a total of 108 terms, with 6 co-occurrences ≥30, which in descending order are hiv, treatment failure, antiretroviral therapy, indinavir and ritonavir. The fifth cluster is blue and contains 84 terms, with 4 co-occurrences ≥30, from highest to lowest being infection, tuberculosis, mycobacterium-tuberculosis, and cord factor.

**FIGURE 10:**
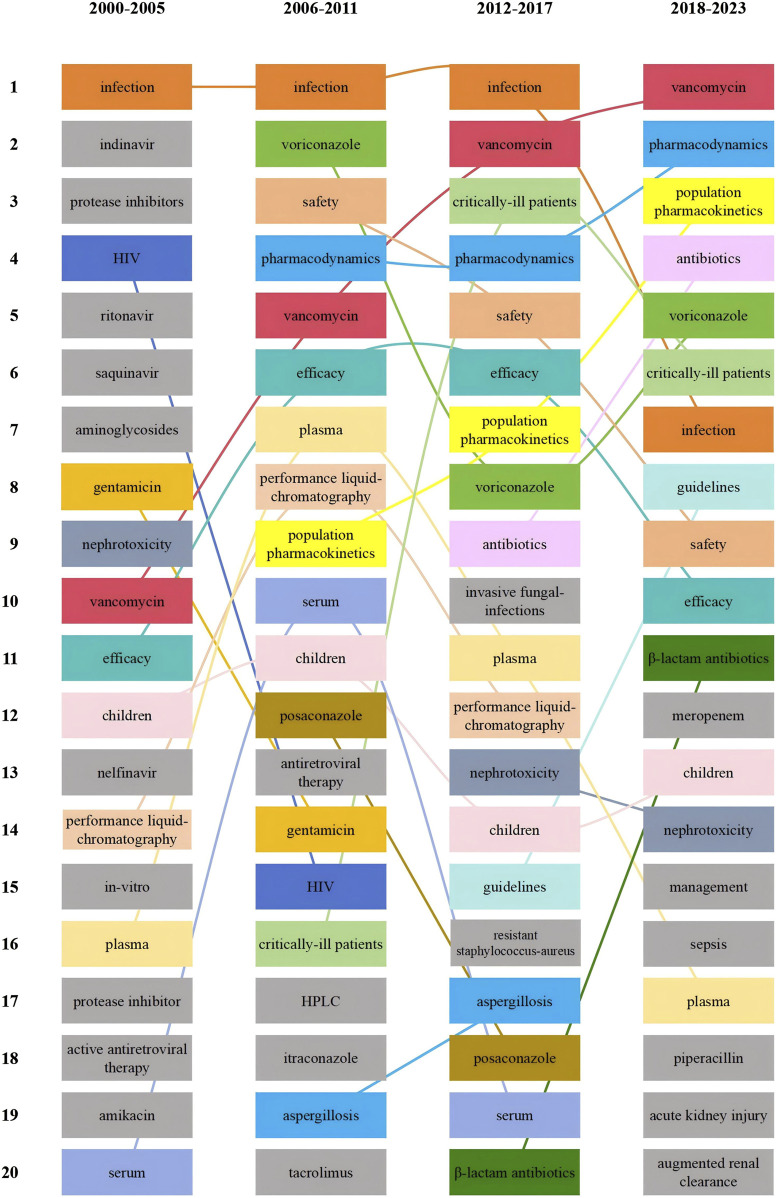
Top 20 hotness rankings of keywords and the changes of ranking of antimicrobial drugs in therapeutic drug monitoring during different time periods.

To demonstrate the changes in research hotspots during different time periods, we divided the included literature into four periods, Figure 10 shows the top 20 hotness rankings of keywords and the changes of rankings during different time periods. Vancomycin has seen an increase in its ranking across the four time periods, eventually overtaking voriconazole in the 2012–2017 period. Additionally, since the 2012–2017 period, critically ill patients entered the top 5.

### 3.6 Co-citation of references and reference bursts

Our analysis of co-cited references revealed that the top 10 were cited a total of 1,053 co-citations and two (Abdul-Aziz et al., 2020; Rybak et al., 2020; Abdul-Aziz et al., 2020; Rybak et al., 2020) had ≥100 co-citations (Table 3). The most co-cited reference was published by Abdul-Aziz MH in *Intensive Care Medicine* in 2020 (Abdul-Aziz et al., 2020).

**TABLE 3:** Top 10 co-cited references for antimicrobials in therapeutic drug monitoring studies.

Rank	Year	Reference	Co-citation
1	2020	Abdul-Aziz MH, et al. Antimicrobial therapeutic drug monitoring in critically ill adult patients: a Position Paper. Intensive Care Med. 2020; 46(6):1127–1153. doi:10.1007/s00134-020-06050-1	223
2	2020	Rybak MJ, Le J, et al. Therapeutic monitoring of vancomycin for serious methicillin-resistant *Staphylococcus aureus* infections: A revised consensus guideline and review by the American Society of Health-System Pharmacists, the Infectious Diseases Society of America, the Pediatric Infectious Diseases Society, and the Society of Infectious Diseases Pharmacists. Am J Health Syst Pharm. 2020; 77(11):835–864. doi:10.1093/ajhp/zxaa036	152
3	2019	Guilhaumou R, et al. Optimization of the treatment with β-lactam antibiotics in critically ill patients-guidelines from the French Society of Pharmacology and Therapeutics (Société Française de Pharmacologie et Thérapeutique-SFPT) and the French Society of Anaesthesia and Intensive Care Medicine (Société Française d'Anesthésie et Réanimation-SFAR). Crit Care. 2019; 23(1):104. doi:10.1186/s13054-019-2378-9	109
4	2018	Neely MN, et al. Prospective Trial on the Use of Trough Concentration versus Area under the Curve To Determine Therapeutic Vancomycin Dosing. Antimicrob Agents Chemother. 2018; 62(2):e02042-17. doi:10.1128/AAC.02042-17	101
5	2014	Ashbee HR, et al. Therapeutic drug monitoring (TDM) of antifungal agents: guidelines from the British Society for Medical Mycology. J Antimicrob Chemother. 2014; 69(5):1162–76. doi:10.1093/jac/dkt508	84
6	2020	Rybak MJ, et al. Therapeutic Monitoring of Vancomycin for Serious Methicillin-resistant *Staphylococcus aureus* Infections: A Revised Consensus Guideline and Review by the American Society of Health-system Pharmacists, the Infectious Diseases Society of America, the Pediatric Infectious Diseases Society, and the Society of Infectious Diseases Pharmacists. Clin Infect Dis. 2020; 71(6):1361–1364. doi:10.1093/cid/ciaa303	83
7	2008	Pascual A, et al. Voriconazole therapeutic drug monitoring in patients with invasive mycoses improves efficacy and safety outcomes. Clin Infect Dis. 2008; 46(2):201–11. doi:10.1086/524669	82
8	2016	Patterson TF, et al. Practice Guidelines for the Diagnosis and Management of Aspergillosis: 2016 Update by the Infectious Diseases Society of America. Clin Infect Dis. 2016; 63(4):e1-e60. doi:10.1093/cid/ciw326	79
9	2018	Ullmann AJ, et al. Diagnosis and management of Aspergillus diseases: executive summary of the 2017 ESCMID-ECMM-ERS guideline. Clin Microbiol Infect. 2018; 24 Suppl 1:e1-e38. doi:10.1016/j.cmi.2018.01.002	70
10	2014	Roberts JA, et al. DALI: defining antibiotic levels in intensive care unit patients: are current β-lactam antibiotic doses sufficient for critically ill patients? Clin Infect Dis. 2014; 58(8):1072–1083. doi:10.1093/cid/ciu027	70

**FIGURE 11:**
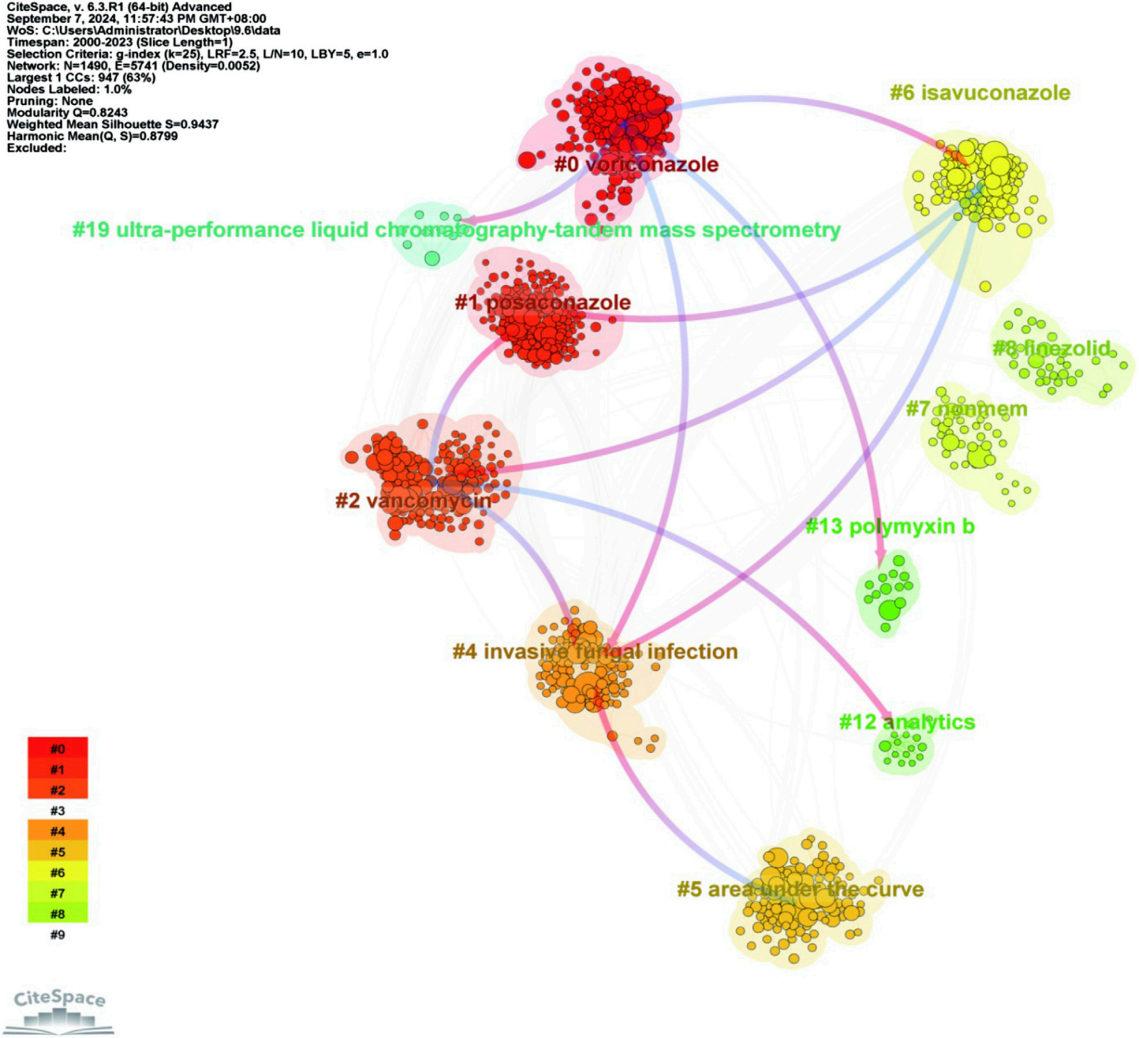
Clustering analysis of keywords in co-cited literature on antimicrobial drugs studied in therapeutic drug monitoring. Note: Node size indicates the co-occurrence frequency of co-cited studies, and different colors indicate different clustering of keywords.

The co-cited literature was analyzed for keyword clustering (Figure 11). Different colors represent the clusters to which different co-cited studies belong and the same colors indicate a close relationship between different co-cited studies. In terms of the connecting lines between clusters, the initial direction (depicted in blue) evolved from the direction of red arrows. As shown in the figure, keyword clustering resulted in division of the co-cited studies into nine categories. The first three clusters were voriconazole, posaconazole and vancomycin.

**FIGURE 12:**
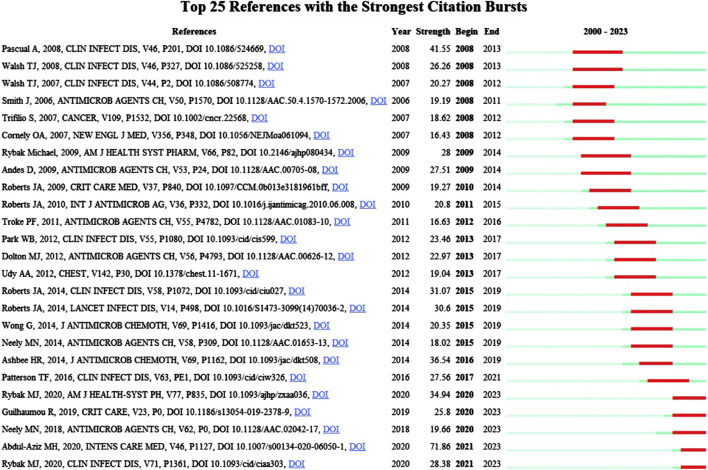
Top 25 references with the strongest citation bursts (sorted by the year the burst began). Note: Blue bars indicate that the reference was published; red bars indicate a citation surge.

CiteSpace co-citation burst analysis was used, with the burst duration of co-cited studies set to a minimum of 2 years. A total of 446 co-citations showed bursts and the studies with the strongest citation bursts were selected (Figure 12). Overall, 24% (6/25) of references showed citation bursts in 2008, followed by 16% (4/25) in 2015. In 2021–2023, “Antimicrobial therapeutic drug monitoring in critically ill adult patients: a Position Paper” (Abdul-Aziz et al., 2020) published in *Intensive Care Medicine* by Abdul-Aziz MH, was the most explosive (strength = 71.86), consistent with results of the co-citation literature analysis. As shown in Table 4, the top 25 co-cited literatures were analyzed according to the dynamics and intensity of the burst, and the research hotspots were identified by analyzing the time trends.

**TABLE 4:** Top 25 antimicrobial research hotspots in therapeutic drug monitoring studies (ranked according to initial year of citation outbreaks in co-cited literature).

Rank	Begin year	Hot research topic	End year	Burst strength
1	2008	*Voriconazole therapeutic monitoring: improving the efficacy and safety of treatment in critically ill patients with invasive fungal disease	2013	41.55
2	2008	Guidelines for the treatment of Aspergillosis: provides standard treatment for Aspergillosis	2013	26.26
3	2008	Posaconazole alternative therapy: provides an alternative for patients who have difficulty tolerating prior antifungal therapy	2012	20.27
4	2008	Voriconazole monitoring in relation to disease progression: drug concentrations are significantly related to disease progression	2011	19.19
5	2008	Voriconazole levels and treatment failure rates: low levels are associated with high failure rates, emphasising the importance of therapeutic drug monitoring	2012	18.62
6	2008	Posaconazole prophylaxis: better than other drugs in preventing invasive fungal infections in certain patient populations	2012	16.43
7	2009	*Vancomycin blood concentration control: emphasis is placed on precise control of blood concentrations to reduce nephrotoxicity and ototoxicity	2014	28
8	2009	Clinical applications of therapeutic drug monitoring: the clinical significance of drug analysis methods, drug dose-exposure relationships, and the relationship between drug concentration and efficacy/toxicity are discussed	2014	27.51
9	2010	*Antibiotic pharmacokinetics: highlighting the importance of understanding the pharmacokinetic properties of antibiotics for the development of personalised dosing regimens	2014	19.27
10	2011	*β-lactam monitoring: assessing its utility in the management of critically ill patients	2015	20.8
11	2012	*Voriconazole drug exposure and clinical response: exploring the relationship between drug exposure and treatment outcome	2016	16.63
12	2013	*Routine TDM with voriconazole: reducing adverse events and improving treatment outcomes	2017	23.46
13	2013	Voriconazole concentrations and clinical outcomes: assessing clinical factors and drug interactions affecting voriconazole concentrations	2017	22.97
14	2013	β-lactam concentrations in critically ill patients: exploring the association between enhanced renal clearance and trough drug concentrations	2017	19.04
15	2015	*Under-exposure to antibiotics and adverse outcomes: highlighting the importance of individualised dosing for the prognosis of critically ill patients	2019	31.07
16	2015	Individualisation of antibiotic dosage in critically ill patients: discussing challenges and solutions	2019	30.6
17	2015	Differences in β-lactam drug testing: exploring differences in test types, patient selection, and drug testing methods	2019	20.35
18	2015	Vancomycin trough concentrations: exploring whether they are sufficient for optimal dosing	2019	18.02
19	2016	*Guidelines for TDM of antifungal drugs: from the British Society for Medical Mycology	2019	36.54
20	2017	*Guidelines for the diagnosis and management of Aspergillosis: providing practical guidelines for the diagnosis and management of Aspergillosis	2021	27.56
21	2020	*Vancomycin efficacy monitoring: revised consensus guidelines	2023	34.94
22	2020	Optimisation of β-lactam therapy in ICU patients: providing guidelines for optimisation	2023	25.8
23	2020	Vancomycin dose optimisation: AUC-guided, Bayesian estimation-assisted dosing correlates with reduced nephrotoxicity, reduced blood sampling, and shorter treatment duration	2023	19.66
24	2021	*Routine TDM in critically ill patients: for a wide range of drugs, including aminoglycosides, β-lactam antibiotics, and more	2023	71.86
25	2021	New consensus on vancomycin administration and monitoring: executive summary provided	2023	28.38

*Research hotspots that represent the highest intensity of outbreaks in the year.

## 4 Discussion

This study used bibliometric and visualization methods to provide a comprehensive analysis of research trends in antimicrobial TDM since the twenty-first century. The number of publications in this field increasing more than 15-fold between 2000 and 2023. Our findings highlight the key research hotspots and potential future research directions in the field of TDM, providing an empirical basis for scientific collaboration and knowledge translation.

Among the 3,710 articles included in this study, there are more types of studies related to clinical, retrospective, PK, and toxicity, and the focus groups are mostly concentrated in critically ill patients, children, and hematology (Figure 3). This may be related to the need for more accurate adjustment of the dosage of antibiotics used in this patient type (Williams et al., 2024; Meesters et al., 2022). PK research is usually in the early stages of drug development and may receive more attention, but the need for PK may be more urgent in clinical practice, such as in personalised medication and dose adjustment (Bhandari et al., 2024; Qin et al., 2022). At the same time, PD research involves complex biological responses and individual differences, but it is difficult to obtain and analyze data, which may lead to a small number of relevant literatures (Moore et al., 2023).

Academic capacity largely depends on the economic status of a country (Kiraz, 2020). The output of medical research serves as an indirect indicator of the degree of national healthcare expenditure (Wu et al., 2021; Wen et al., 2011; Meo et al., 2013). Europe is the continent with the highest number of countries conducting research. The United States had the highest number of articles published in this field (a total of 794), followed by China, Australia, France, Italy, Japan, and the Netherlands. Four countries (the United States, France, England, and Germany) had high median centrality (≥0.1), which is considered a key turning point leading to transformative findings. This information provides researchers worldwide with potential partners and institutions for collaboration and facilitates cross-border, interdisciplinary research alliances.

The top drug-related keywords, such as vancomycin, voriconazole, and meropenem, representing the current mainstream antimicrobial drugs under TDM. The high frequency of articles on these drugs suggests ongoing efforts to refine their use, including TDM strategies to optimize dosing and minimize toxicity. Vancomycin, in particular, has seen an increase in research attention, likely due to its critical role in treating serious Gram-positive infections and the challenges associated with achieving therapeutic concentrations in certain patient populations (Chang et al., 2023).

The proximity and prevalence of research topics in scientific fields are revealed by co-occurrence analysis of keywords (Deng et al., 2020). Keyword co-occurrence and emerging item analyses facilitates the identification of hot topics in a given field over time and the clustering of keywords provided by the authors in the dataset. In this study, the main high-frequency keywords included therapeutic drug monitoring, pharmacokinetics, vancomycin, pharmacodynamics, voriconazole, population pharmacokinetics, antibiotics, safety, critically ill patients, and infections. Keyword clustering describes the internal knowledge structure of a research area and categorizes its domain. The findings indicate that TDM and pharmacokinetic evaluation of vancomycin are research hotspots for avoiding adverse reactions and achieving optimal clinical outcomes, and have been recommended by guidelines (He et al., 2020; Rybak et al., 2020).

In the keyword cluster analysis plot of co-cited literature (Figure 11), the clusters highlighted by the red arrows represented the origins giving rise to a new cluster, while the blue initial directions were new clusters derived through evolution. Based on the findings, it is hypothesized that voriconazole, vancomycin, and isavuconazole are emerging significant antimicrobial drugs in the field of TDM. In future studies, further attention needs to be paid to the toxicity of the above drugs and careful consideration of antimicrobials in terms of invasive fungal infections.

A hotspot is a scientific theme in a particular research area that has emerged over a period of time and serves as one of the fundamental methods of bibliometric analysis (Wu et al., 2021c). As illustrated in Table 4, voriconazole emerged as the predominant research hotspot during the period from 2008 to 2013. Subsequently, critical patients and vancomycin gained prominence as new research hotspots from 2015 to 2021. This consistency aligns with the shifts in keyword rankings across various time periods (Figure 10), indicating that vancomycin and critically ill patients have emerged as the latest research hotspots. For vancomycin, a significant increase in the number of measurements was recorded (Voulgaridou et al., 2023). Multivariate analysis indicated that being overweight and experiencing vancomycin-induced nephrotoxicity were independent risk factors for higher all-cause mortality, appropriate TDM is a crucial strategy to enhance treatment efficacy and mitigate the severe toxicity (Al-Maqbali et al., 2022).

In summary, critically ill patients, children, and hematology patients are currently the main patient groups undergoing TDM in clinical practice (Cojutti et al., 2023). In clinical practice, TDM should be a key consideration when patients need to use drugs such as vancomycin, voriconazole, meropenem, posaconazole, gentamicin, etc, and further studies can be carried out through the key journals and co-cited literatures provided in this paper. In other patient types (the elderly, obesity, other diseases, etc.) facing the use of antibiotics with high frequency of the keywords in this paper, the awareness of the use of TDM should be raised, and this direction has new research potential. In terms of the type of research, it may indicate future research opportunities in the PD field, especially in the clinical application of drugs and personalized therapy.

The current study has several limitations that should be taken into consideration. Firstly, data were retrieved solely from WOSCC. While WOS is recommended as the most reliable database for bibliometric studies, some articles may have been overlooked. To overcome this limitation, we also conducted bibliometric analysis using the PubMed database, and the results are shown in Supplementary Table S1, Supplementary Figures S1–S4. We found that there was no significant difference in the trend of the research results obtained from the two databases, only a difference in quantity. Secondly, the majority of articles were published in English and this choice of language restriction could lead to bias. Thirdly, there may be some inconsistencies in various aspects, for, e.g., an organization could use different names at various times, and other possible scenarios.

## 5 Conclusion

The significant increase in the number of annual publications reflects the importance of TDM for antimicrobials. This study identifies the major researchers, institutions, Key words, co-cited literature involved in research related to antimicrobials in TDM on a global scale, and the trend over time. The clinical significance of this research in improving efficacy and reducing toxicity in a variety of clinical populations was also discussed. *Therapeutic Drug Monitoring* is the most prolific journal in this field. Vancomycin, voriconazole, children and critically ill patients are the current hot topics, while vancomycin, voriconazole, meropenem, isavuconazole, posaconazole, teicoplanin, children and critically ill patients are the focus of future research. The collective findings provide researchers and policymakers with a comprehensive overview of the broader landscape.

## Data Availability

The original contributions presented in the study are included in the article/Supplementary Material, further inquiries can be directed to the corresponding author.
