# Supplementary material for: Global research trends in therapeutic drug monitoring of antimicrobials from 2000 to 2023: a bibliometric analysis
Source: Front Pharmacol. 2024 Dec 3;15:1474878. doi: 10.3389/fphar.2024.1474878 (PMC11649425; doi:10.3389/fphar.2024.1474878)
Supplement: Supplementary file 1 [file DataSheet1.docx]

The Pubmed database advanced search entry is set as the following: (("Agents, Anti-Bacterial"[Title/Abstract]) OR ("Anti Bacterial Agents"[Title/Abstract]) OR ("Anti-Bacterial Compounds"[Title/Abstract]) OR ("Anti Bacterial Compounds"[Title/Abstract]) OR ("Compounds, Anti-Bacterial"[Title/Abstract]) OR ("Antibacterial Agents"[Title/Abstract]) OR ("Agents, Antibacterial"[Title/Abstract]) OR ("Anti-Bacterial Agent"[Title/Abstract]) OR ("Agent, Anti-Bacterial"[Title/Abstract]) OR ("Anti Bacterial Agent"[Title/Abstract]) OR ("Anti-Bacterial Compound"[Title/Abstract]) OR ("Anti Bacterial Compound"[Title/Abstract]) OR ("Compound, Anti-Bacterial"[Title/Abstract]) OR ("Antibacterial Agent"[Title/Abstract]) OR ("Agent, Antibacterial"[Title/Abstract]) OR ("Antibiotics"[Title/Abstract]) OR ("Antibiotic"[Title/Abstract]) OR ("Anti-Mycobacterial Agents"[Title/Abstract]) OR ("Agents, Anti-Mycobacterial"[Title/Abstract]) OR ("Anti Mycobacterial Agents"[Title/Abstract]) OR ("Antimycobacterial Agents"[Title/Abstract]) OR ("Agents, Antimycobacterial"[Title/Abstract]) OR ("Anti-Mycobacterial Agent"[Title/Abstract]) OR ("Agent, Anti-Mycobacterial"[Title/Abstract]) OR ("Anti Mycobacterial Agent"[Title/Abstract]) OR ("Antimycobacterial Agent"[Title/Abstract]) OR ("Agent, Antimycobacterial"[Title/Abstract]) OR ("Bacteriocidal Agents"[Title/Abstract]) OR ("Agents, Bacteriocidal"[Title/Abstract]) OR ("Bacteriocides"[Title/Abstract]) OR ("Bacteriocidal Agent"[Title/Abstract]) OR ("Agent, Bacteriocidal"[Title/Abstract]) OR ("Bacteriocide"[Title/Abstract]) OR ("meropenem"[Title/Abstract]) OR ("vancomycin"[Title/Abstract]) OR ("piperacillin"[Title/Abstract]) OR ("ceftazidime"[Title/Abstract]) OR ("cefepime"[Title/Abstract]) OR ("gentamicin"[Title/Abstract]) OR ("tazobactam"[Title/Abstract]) OR ("piperacillin-tazobactam"[Title/Abstract]) OR ("ciprofloxacin"[Title/Abstract]) OR ("amikacin"[Title/Abstract]) OR ("ceftriaxone"[Title/Abstract]) OR ("daptomycin"[Title/Abstract]) OR ("ertapenem"[Title/Abstract]) OR ("imipenem"[Title/Abstract]) OR ("moxifloxacin"[Title/Abstract]) OR ("voriconazole"[Title/Abstract]) OR ("colistin"[Title/Abstract]) OR ("flucloxacillin"[Title/Abstract]) OR ("tobramycin"[Title/Abstract]) OR ("clarithromycin"[Title/Abstract]) OR ("amoxicillin"[Title/Abstract]) OR ("cilastatin"[Title/Abstract]) OR ("levofloxacin"[Title/Abstract]) OR ("polymyxin-b"[Title/Abstract]) OR ("teicoplanin"[Title/Abstract]) OR ("trimethoprim-sulfamethoxazole"[Title/Abstract]) OR ("adriamycin"[Title/Abstract]) OR ("amphotericin-b"[Title/Abstract]) OR ("ampicillin"[Title/Abstract]) OR ("cefotaxime"[Title/Abstract]) OR ("carbapenems"[Title/Abstract]) OR ("caspofungin"[Title/Abstract]) OR ("cefazolin"[Title/Abstract]) OR ("meropenem"[Title/Abstract]) OR ("vancomycin"[Title/Abstract]) OR ("piperacillin"[Title/Abstract]) OR ("ceftazidime"[Title/Abstract]) OR ("cefepime"[Title/Abstract]) OR ("gentamicin"[Title/Abstract]) OR ("tazobactam"[Title/Abstract]) OR ("piperacillin-tazobactam"[Title/Abstract]) OR ("ciprofloxacin"[Title/Abstract]) OR ("amikacin"[Title/Abstract]) OR ("ceftriaxone"[Title/Abstract]) OR ("daptomycin"[Title/Abstract]) OR ("ertapenem"[Title/Abstract]) OR ("imipenem"[Title/Abstract]) OR ("moxifloxacin"[Title/Abstract]) OR ("voriconazole"[Title/Abstract]) OR ("colistin"[Title/Abstract]) OR ("flucloxacillin"[Title/Abstract]) OR ("tobramycin"[Title/Abstract]) OR ("clarithromycin"[Title/Abstract]) OR ("amoxicillin"[Title/Abstract]) OR ("cilastatin"[Title/Abstract]) OR ("levofloxacin"[Title/Abstract]) OR ("polymyxin-b"[Title/Abstract]) OR ("teicoplanin"[Title/Abstract]) OR ("trimethoprim-sulfamethoxazole"[Title/Abstract]) OR ("adriamycin"[Title/Abstract]) OR ("amphotericin-b"[Title/Abstract]) OR ("ampicillin"[Title/Abstract]) OR ("cefotaxime"[Title/Abstract]) OR ("carbapenems"[Title/Abstract]) OR ("caspofungin"[Title/Abstract]) OR ("cefazolin"[Title/Abstract]) OR ("infection"[Title/Abstract]) OR ("infections"[Title/Abstract])) AND (("TDM"[Title/Abstract]) OR ("Drug Monitoring"[Title/Abstract]) OR ("Monitoring Drug"[Title/Abstract]) OR ("Therapeutic Drug Monitoring"[Title/Abstract]) OR ("Drug Monitoring Therapeutic"[Title/Abstract]) OR ("Monitoring Therapeutic Drug"[Title/Abstract]) OR ("target concentration intervention"[Title]) OR ("target concentration strategy"[Title]) OR ("model-based precision dosing"[Title])). The search yielded 3514 documents (limits: 2000-2023).

**Table S1** Top 10 journals on antimicrobials in therapeutic drug monitoring research.

| **Rank** | **Journal** | **Count (%)** | **IF (2023)** | **JCR** |
| --- | --- | --- | --- | --- |
| 1 | Therapeutic Drug Monitoring | 175 (4.98%) | 2.8 | Q2 |
| 2 | Antimicrobial Agents and Chemotherapy | 153 (4.35%) | 4.1 | Q1 |
| 3 | Journal of Antimicrobial Chemotherapy | 126 (3.59%) | 3.9 | Q1 |
| 4 | International Journal of Antimicrobial Agents | 109 (3.10%) | 4.9 | Q1 |
| 5 | Antibiotics-Basel | 97 (2.76%) | 4.3 | Q1 |
| 6 | Journal of Pharmaceutical and Biomedical Analysis | 63 (1.79%) | 3.1 | Q2 |
| 7 | Frontiers in Pharmacology | 55 (1.57%) | 4.4 | Q1 |
| 8 | Journal of Chromatography B-Analytical Technologies in The Biomedical and Life Sciences | 51 (1.45%) | 2.8 | Q2 |
| 9 | Pharmacotherapy | 51 (1.45%) | 2.9 | Q2 |
| 10 | Journal of Infection and Chemotherapy | 46 (1.31%) | 1.9 | Q3 |

**Figure legends**

**Figure S1.** Trends in the number of publications from the twenty-first century in studies of therapeutic drug monitoring for antimicrobial drug applications.

**Figure S2.** Distribution of publications on antimicrobials in therapeutic drug monitoring research: country collaborative network map (A) and top 20 countries (B).

**Figure S3.** Distribution of publications on antimicrobials in therapeutic drug monitoring research: a network map of institutional collaborations (A) and the top 20 institutions(B).

**Figure S4.** Co-occurrence network of antimicrobial keywords in therapeutic drug monitoring.


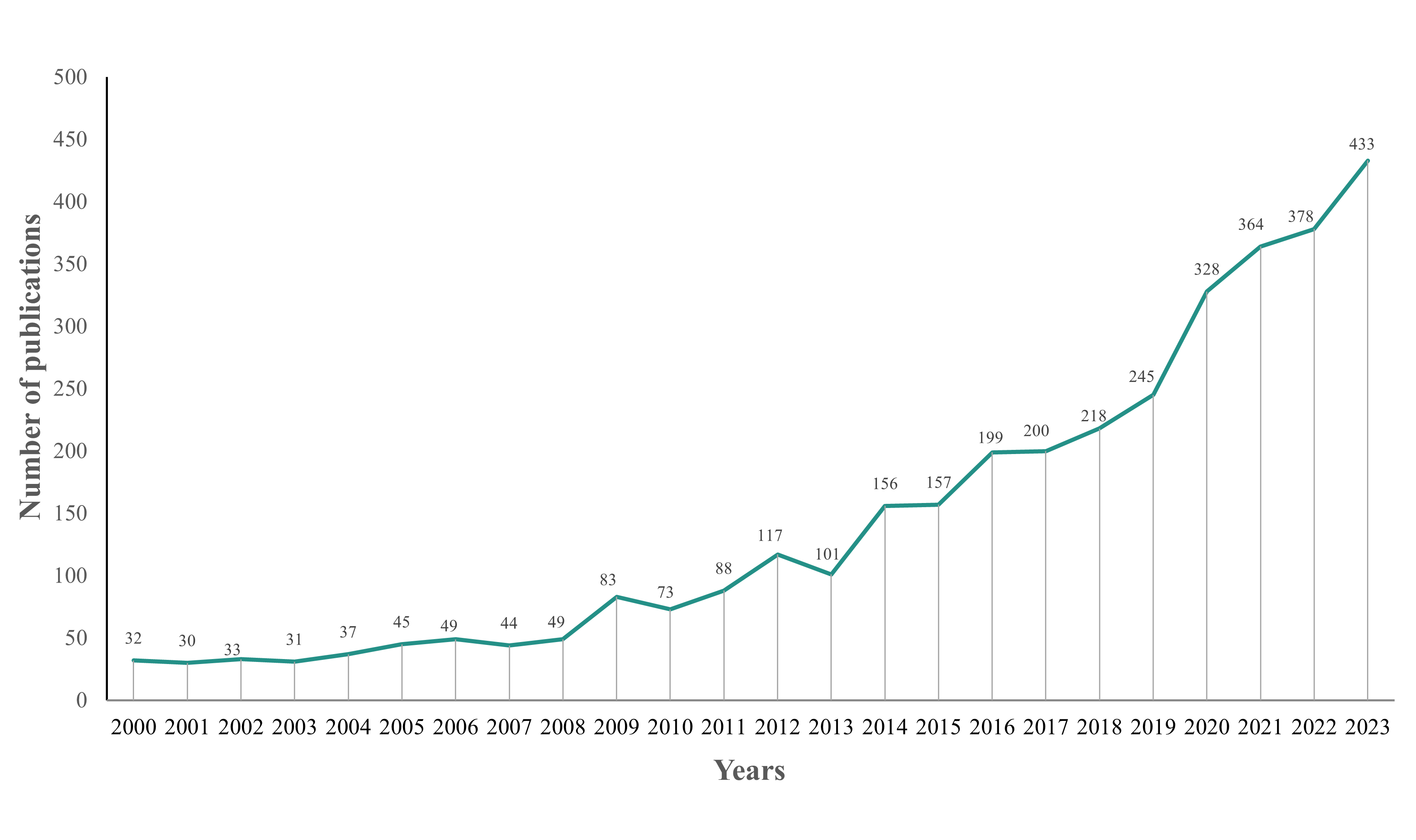


**Figure S1.** Trends in the number of publications from the twenty-first century in studies of therapeutic drug monitoring for antimicrobial drug applications.


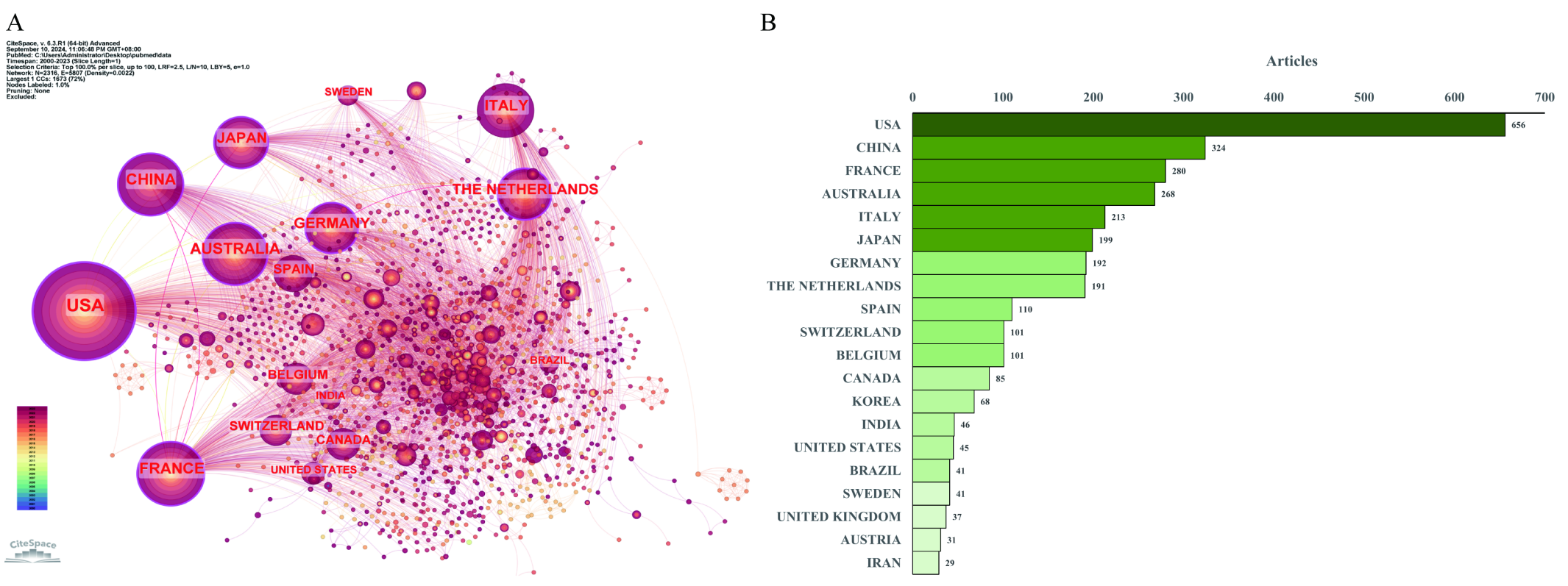


**Figure S2.** Distribution of publications on antimicrobials in therapeutic drug monitoring research: country collaborative network map (A) and top 20 countries (B).


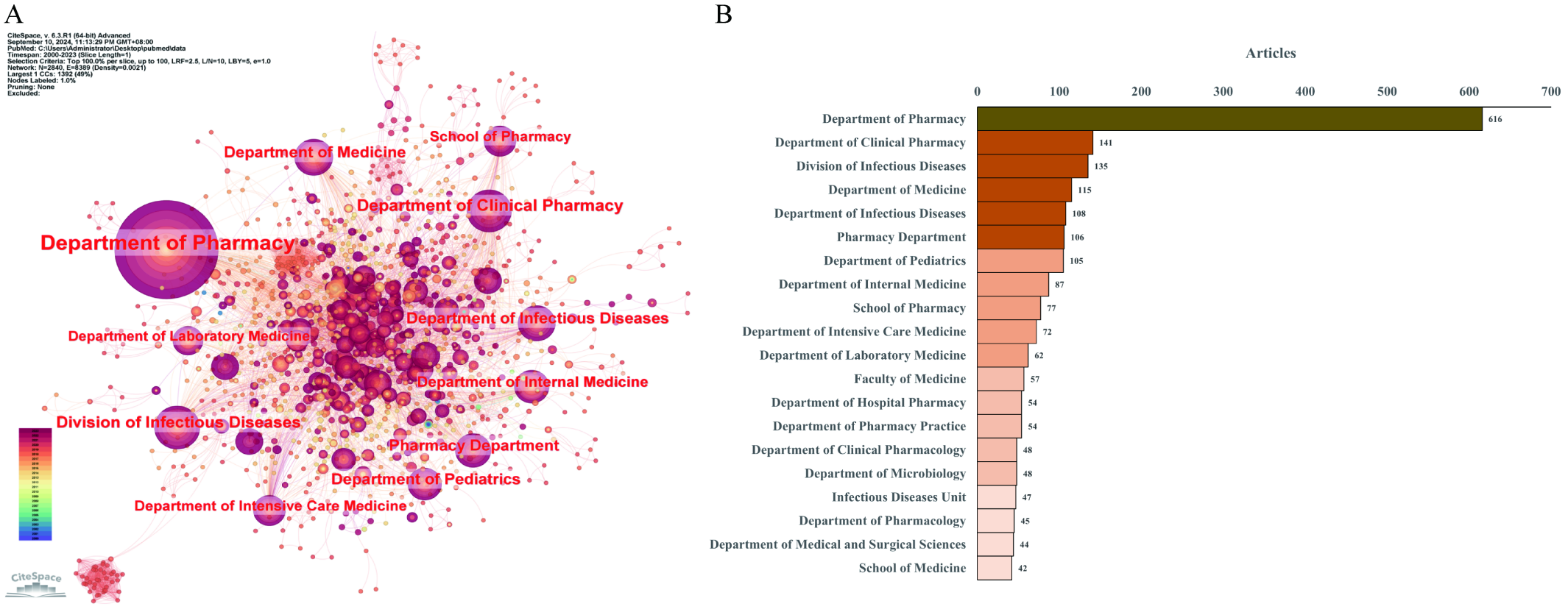


**Figure S3.** Distribution of publications on antimicrobials in therapeutic drug monitoring research: a network map of institutional collaborations (A) and the top 20 institutions(B).


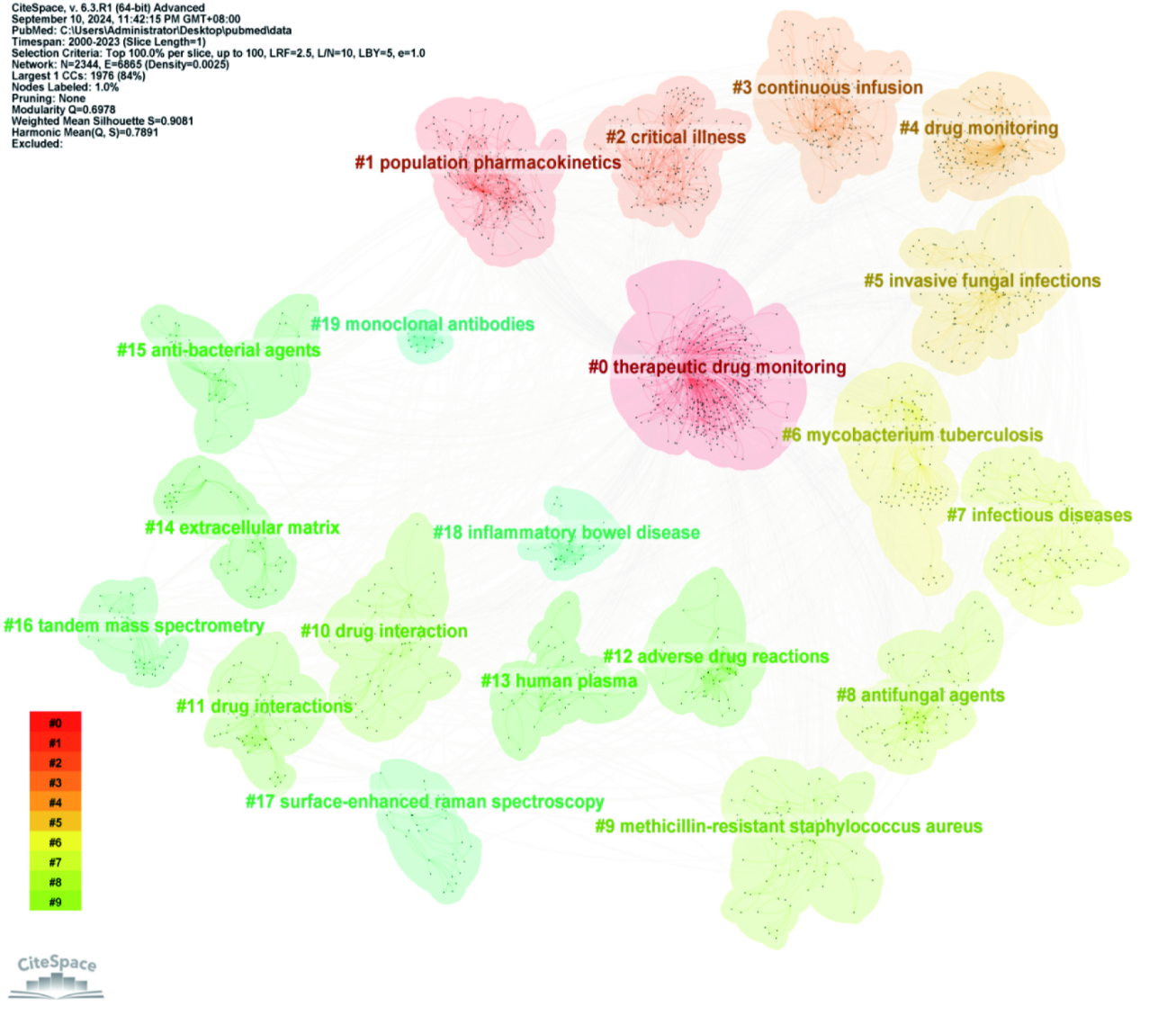


**Figure S4.** Co-occurrence network of antimicrobial keywords in therapeutic drug monitoring.
